# Melatonin and Glutathione Co-Supplementation Improves Redox Homeostasis and Developmental Competence in Sheep Oocytes

**DOI:** 10.3390/biology15201858

**Published:** 2026-10-15

**Authors:** Xiunan Li, Jiajia Zhang, Lede Su, Daqing Wang, Xingyu Zhang, Guoqiang Yuan, Yanyan Yang, Guifang Cao

**Affiliations:** 1 College of Veterinary Medicine, Inner Mongolia Agricultural University, Hohhot 010011, China; lixiunan2368@163.com (X.L.); zhangjiajia@emails.imau.edu.cn (J.Z.); wangdaqing050789@126.com (D.W.); 17725760839@163.com (X.Z.); 13296936866@163.com (G.Y.); 2 Inner Mongolia Academy of Agricultural and Animal Husbandry Sciences, Hohhot 010011, China; susulide@126.com; 3 Animal Embryo and Developmental Engineering Key Laboratory of Higher Education, Institutions of InnerMongolia Autonomous Region, Hohhot 010011, China; 4 Inner Mongolia Autonomous Region Key Laboratory of Basic Veterinary Medicine, Hohhot 010011, China; 5 College of Life Sciences, Inner Mongolia University, Hohhot 010021, China

**Keywords:** melatonin, glutathione, in vitro maturation, oxidative stress, Nrf2, in vitro embryo culture

## Abstract

Oxidative stress is a major factor limiting oocyte quality during in vitro maturation (IVM) and subsequent embryonic developmental competence. This study investigated the effects of combined melatonin (MLT) and glutathione (GSH) supplementation on sheep oocyte quality and the potential regulatory mechanisms involved. Sheep oocytes were assigned to Control, MLT, GSH, and MLT+GSH groups, and oocyte maturation, redox homeostasis, and mitochondrial function were evaluated by assessing cumulus expansion, first polar body extrusion, F-actin organization, ROS- and GSH-associated fluorescence, JC-1 staining, and MitoTracker signals, while subsequent developmental competence was assessed following in vitro fertilization (IVF). SMART-seq2 transcriptomic analysis, Nrf2 immunofluorescence, antioxidant-related gene expression analysis, and pharmacological inhibition of Nrf2 with ML385 were further performed to explore the underlying mechanisms. MLT and GSH, either alone or in combination, improved multiple indices related to oocyte maturation, redox status, and mitochondrial function, with the MLT+GSH group generally showing the most favorable numerical changes. MLT+GSH treatment was associated with greater cumulus expansion, a higher first polar body extrusion rate, improved F-actin organization, reduced ROS accumulation, increased GSH-associated fluorescence, a higher JC-1 red-to-green fluorescence ratio, and enhanced mitochondrial-associated fluorescence. However, for some endpoints, the numerical improvement observed with MLT+GSH did not differ significantly from the corresponding single treatment. Following IVF, MLT+GSH treatment increased cleavage and blastocyst formation rates and improved subsequent developmental outcomes. Mechanistically, MLT+GSH treatment was accompanied by increased Nrf2 immunofluorescence intensity and altered expression of NFE2L2 and several Nrf2-related antioxidant genes, including NQO1, HMOX1, GCLC, GCLM, and GPX4, suggesting the involvement of an Nrf2-associated antioxidant regulatory network. Addition of the Nrf2 inhibitor ML385 to the MLT+GSH treatment increased ROS levels and reduced JC-1 and other redox- and mitochondria-related indices, while partially attenuating improvements in oocyte maturation and embryonic development. Overall, MLT and GSH co-supplementation was associated with improved redox homeostasis, antioxidant capacity, mitochondrial function, and subsequent developmental competence in sheep oocytes, with Nrf2-associated antioxidant regulation potentially contributing to these beneficial effects under standard IVM conditions.

## Simple Summary

Oocyte quality is a key determinant of in vitro embryo production efficiency and reproductive potential in livestock, whereas oxidative stress-induced redox imbalance and mitochondrial dysfunction during in vitro maturation (IVM) can markedly compromise oocyte developmental competence. This study aimed to investigate the effects of combined melatonin (MLT) and glutathione (GSH) supplementation on the developmental competence of sheep oocytes and to explore the potential antioxidant mechanisms involved. The results showed that MLT+GSH co-treatment promoted cumulus expansion and first polar body extrusion, reduced oxidative stress, increased intracellular GSH levels, and improved mitochondrial function. Combined supplementation also enhanced subsequent embryonic development following in vitro fertilization, as reflected by increased cleavage and blastocyst formation rates. Further analyses revealed that MLT+GSH treatment was accompanied by enhanced Nrf2-associated antioxidant responses and altered expression of antioxidant-related genes, including NFE2L2, NQO1, HMOX1, GCLC, GCLM, and GPX4. Pharmacological inhibition of Nrf2 activity with ML385 partially attenuated some of the improvements in redox homeostasis and developmental competence associated with MLT+GSH treatment, suggesting that Nrf2-related antioxidant pathways may contribute to the protective effects of combined supplementation. Collectively, MLT and GSH co-supplementation improves redox homeostasis, antioxidant capacity, and developmental competence in sheep oocytes, providing a potential strategy for optimizing livestock IVM systems and improving in vitro embryo production efficiency.

## 1. Introduction

Oocyte developmental competence is progressively established during maturation and directly determines the ability of an oocyte to undergo successful fertilization and support subsequent embryonic development [1]. In vitro maturation (IVM) is therefore a critical step in livestock in vitro embryo production. However, once cumulus–oocyte complexes are removed from the physiological follicular microenvironment, they lose the dynamic support provided by follicular fluid and surrounding somatic cells, making oocytes more susceptible to metabolic and oxidative stress [2]. Physiological levels of reactive oxygen species (ROS) participate in meiotic regulation and intracellular signaling, whereas excessive ROS accumulation disrupts redox homeostasis, impairs mitochondrial function and cytoskeletal organization, and ultimately compromises oocyte maturation and subsequent embryonic development [3]. Maintaining an appropriate redox environment during IVM is therefore essential for preserving oocyte quality and developmental competence.

Melatonin (MLT) and glutathione (GSH) are important components of cellular antioxidant defense. MLT possesses potent antioxidant properties and can alleviate oxidative damage in oocytes by scavenging reactive species, modulating endogenous antioxidant responses, and maintaining mitochondrial homeostasis [4,5]. GSH, a major intracellular low-molecular-weight thiol, plays an essential role in maintaining cellular redox balance and supporting glutathione-dependent peroxide detoxification [6]. Adequate intracellular GSH levels are closely associated with oocyte maturation, fertilization, and early embryonic development [7]. Given their distinct roles in antioxidant defense, combined supplementation with MLT and GSH may help maintain a more favorable redox environment during oocyte IVM”. However, the effects of combined MLT and GSH supplementation on sheep oocyte maturation, mitochondrial function, and subsequent embryonic development remain insufficiently characterized, and the underlying molecular regulatory mechanisms require further investigation.

Nuclear factor erythroid 2-related factor 2 (Nrf2), encoded by NFE2L2, is an important transcriptional regulator involved in cellular adaptation to oxidative stress and antioxidant defense [8]. Nrf2-associated antioxidant responses involve multiple genes related to cellular detoxification, glutathione metabolism, and protection against oxidative damage, including NQO1, HMOX1, GCLC, GCLM, and GPX4 [9]. Previous studies have implicated Nrf2-related regulation in maintaining oocyte redox homeostasis and developmental competence [10]; however, whether Nrf2-associated signaling contributes to the response of sheep oocytes to combined MLT and GSH supplementation remains unclear. Therefore, integrating transcriptomic profiling with the assessment of Nrf2-related responses and downstream antioxidant gene expression, together with pharmacological intervention, may provide further insight into the molecular basis underlying the effects of combined supplementation on oocyte redox homeostasis [11].

Accordingly, the present study systematically evaluated the effects of MLT and GSH supplementation, individually and in combination, on sheep oocyte maturation and subsequent developmental competence. Oocyte maturation, cytoskeletal integrity, redox homeostasis, mitochondrial function, and embryonic development following in vitro fertilization were comprehensively assessed. SMART-seq2 transcriptomic profiling was further performed to identify molecular changes associated with combined supplementation, followed by Nrf2 immunofluorescence analysis, antioxidant-related gene expression assessment, and pharmacological intervention with ML385 to evaluate the potential contribution of Nrf2-associated antioxidant responses to the observed beneficial effects under standard IVM conditions. This study aimed to provide new insights into the biological basis by which combined MLT and GSH supplementation improves sheep oocyte developmental competence and to provide a theoretical basis for optimizing livestock IVM and in vitro embryo production systems.

## 2. Materials and Methods

### 2.1. Materials and Equipment

The following reagents were used: IVM Medium (REF 1.03.020), IVC Medium (REF 1.07.020), and OPU Medium (REF 2.01.500) from Stroebech Medi (Copenhagen, Denmark); hyaluronidase (H4272), ML385 (SML1833, St. Louis, MO, USA), mineral oil (M8410), and BODIPY 493/503 (D3922) from Sigma-Aldrich (St. Louis, MO, USA) or Invitrogen (Carlsbad, CA, USA), as specified by the manufacturer (USA); Medium 199 (11150059), Dulbecco’s phosphate-buffered saline (DPBS; C3590-0500), and penicillin-streptomycin (C3420-0100) from Gibco (Grand Island, NY, USA); an ROS assay kit (S0035S), JC-1 staining solution (C2003S), Mito-Tracker Red CMXRos (C1035), an Nrf2 rabbit polyclonal antibody (AF7623), an anti-rabbit Alexa Fluor 488 immunofluorescence kit (P0176), and Actin-Tracker Green-488 (C2201S) from Beyotime (Shanghai, China); CellTracker Blue CMF2HC Dye (215868-45-4) from MedChemExpress MedChemExpress (Monmouth Junction, NJ, USA); and an Annexin V-FITC Apoptosis Detection Kit (V13242) from Invitrogen (Carlsbad, CA, USA). Reagents were stored and used according to the manufacturers’ instructions.

Major equipment included an electrophoresis system (Bio-Rad, Hercules, CA, USA), pipettes (Eppendorf, Hamburg, Germany), a CO_2_ incubator (Thermo Fisher Scientific, Waltham, MA, USA), centrifuges (Himac, Tokyo, Japan) and Thermo Fisher Scientific, Waltham, MA, USA), an Olympus microscopy imaging system (Olympus, Tokyo, Japan), and a laser-scanning confocal microscope (ZEISS, Oberkochen, Germany). Solutions requiring sterilization were passed through syringe filters, and experimental instruments were sterilized before use.

### 2.2. Experimental Groups

The concentrations of MLT and GSH were selected primarily based on previous studies concerning oocyte in vitro maturation, redox homeostasis, and embryonic development, and were adapted to the present in vitro culture system. The final working concentrations were 10^−9^ mol/L (1 nM) for MLT and 2 mmol/L for GSH. The 1 nM concentration of MLT represents a relatively low effective concentration previously used in in vitro oocyte culture studies and was selected based on evidence from in vitro systems rather than by directly extrapolating circulating drug concentrations in ruminants. The working concentration of 2 mmol/L GSH was likewise determined with reference to previous related studies. To further evaluate the potential involvement of Nrf2-related regulation in the effects of MLT+GSH treatment, 5 μmol/L ML385 was used as a pharmacological inhibitor based on previous studies; the ML385 dose–response curve is provided in Supplementary Table S1. ML385 was prepared as a 10 mmol/L stock solution in DMSO and diluted to a final concentration of 5 μmol/L for treatment, resulting in a final DMSO concentration of 0.05% (*v*/*v*) in the culture medium. To exclude potential solvent-related effects, a matched DMSO vehicle control was included in the ML385 intervention experiment. Accordingly, the Control group in Section 3.6 received 0.05% DMSO, whereas the Control group in Section 3.1, Section 3.2, Section 3.3, Section 3.4 and Section 3.5 received no exogenous additives. The detailed treatment conditions for each experimental group are shown in Table 1. For consistency, the group codes I and MGI are used throughout the manuscript and figures to represent the ML385 and MLT+GSH+ML385 groups, respectively.

### 2.3. In Vitro Maturation and Fertilization of Sheep Oocytes

Sheep ovaries were collected under aseptic conditions and washed after removal of excess surface tissue. Cumulus-oocyte complexes (COCs) were released by slicing the ovarian cortex. COCs surrounded by at least three compact layers of cumulus cells and containing homogeneous cytoplasm were selected, sequentially washed in OPU, HM, and IVM media, and randomly assigned to treatment groups for 24 h of IVM. MLT, GSH, and ML385 were added according to the experimental design. After maturation, cumulus cells were removed by hyaluronidase treatment, and the denuded oocytes were subjected to in vitro fertilization. Thawed semen was washed and centrifuged, adjusted to approximately 1 × 10^6^ spermatozoa/mL, and co-incubated with oocytes in IVF droplets for 22–26 h. Presumptive zygotes were then transferred to embryo culture medium. Cleavage rate, blastocyst formation rate, and blastocyst quality were evaluated.

### 2.4. Assessment of Cumulus Expansion and First Polar Body Extrusion

At the end of IVM, cumulus expansion was scored under an inverted microscope using a four-point scale (0–3): score 0, no expansion; score 1, expansion confined to the outer cumulus-cell layer; score 2, clear expansion of the outer and middle layers with a compact inner layer; and score 3, complete expansion with loosely distributed cumulus cells throughout all layers. At least 30 randomly selected COCs per group were evaluated in each biological replicate. The mean expansion score and the proportion of highly expanded COCs were calculated by an investigator blinded to group allocation. Cumulus cells were subsequently removed with 0.1% hyaluronidase. Oocytes with a clearly visible first polar body in the perivitelline space were classified as metaphase II (MII) oocytes, and the first polar body extrusion rate was calculated.

### 2.5. Assessment of Redox and Metabolic Indices

#### 2.5.1. ROS- and GSH-Associated Fluorescence

Denuded mature oocytes were washed in PBS and incubated separately with 5 μM CM-H2DCFDA or 5 μM CellTracker Blue CMF2HC Dye for 30 min at 37 °C in the dark, followed by three washes in PBS. Fluorescence was recorded using a laser-scanning confocal microscope. CM-H2DCFDA-derived green fluorescence was detected at excitation/emission wavelengths of 495/530 nm as an index of intracellular ROS. CellTracker Blue-derived fluorescence was detected at excitation/emission wavelengths of 450/460 nm as an index of GSH-associated fluorescence. All images were acquired using identical exposure and imaging settings, and fluorescence intensity was quantified.

#### 2.5.2. Mitochondrial Membrane Potential

Mitochondrial membrane potential was assessed using JC-1 staining. Denuded mature oocytes were washed twice in PBS, transferred to pre-warmed JC-1 working solution, and incubated for 20 min at 37 °C in the dark. After three washes in PBS, fluorescence was recorded by confocal microscopy. Green fluorescence from JC-1 monomers was collected at excitation/emission wavelengths of 514/529 nm, and red fluorescence from JC-1 aggregates was collected at 585/590 nm. Mitochondrial membrane potential was expressed as the ratio of red to green fluorescence intensity.

#### 2.5.3. Assessment of Mitochondrial Distribution

Mitochondrial-associated fluorescence was assessed using Mito-Tracker Red CMXRos (C1035; Beyotime, China). Denuded mature oocytes were washed twice in PBS and incubated in freshly prepared Mito-Tracker Red CMXRos working solution for 30 min at 37 °C in the dark. After three washes in PBS, red fluorescence was recorded by confocal microscopy at excitation/emission wavelengths of 579/599 nm. Fluorescence intensity and distribution were analyzed as indices of mitochondrial status.

#### 2.5.4. Lipid-Droplet Staining

Intracellular lipid droplets were detected using BODIPY 493/503. Denuded mature oocytes were washed twice in PBS and incubated in BODIPY 493/503 working solution for 30 min at 37 °C in the dark. After three washes in PBS to remove unbound dye, fluorescence images were acquired by confocal microscopy at an excitation wavelength of 488 nm and an emission range of 510–530 nm. Green fluorescence intensity was quantified to compare lipid-droplet-associated signals among groups. BODIPY fluorescence was quantified as the background-corrected mean fluorescence intensity within the whole-oocyte region of interest (ROI), thereby minimizing the influence of differences in the analyzed area among oocytes.

### 2.6. F-Actin Staining

The F-actin cytoskeleton was stained with Actin-Tracker Green-488 (C2201S; Beyotime, China). Denuded oocytes collected after IVM were washed in PBS and incubated in Actin-Tracker Green-488 working solution for 30 min at 37 °C in the dark. After three gentle washes in PBS, green fluorescence was recorded by confocal microscopy at an excitation wavelength of 488 nm and an emission range of 500–550 nm. F-actin fluorescence intensity and cortical distribution were analyzed to assess cytoskeletal organization.

### 2.7. TUNEL Assay in Blastocysts

Blastocysts from each group were washed in PBS and fixed, permeabilized, and fluorescently labeled according to the instructions of the TUNEL assay kit. Nuclei were counterstained with DAPI. Confocal microscopy was used to record TUNEL-positive red fluorescence and DAPI-positive blue fluorescence. Apoptosis was evaluated as the proportion of TUNEL-positive nuclei relative to the total number of nuclei.

### 2.8. Immunofluorescence Detection and Quantification of Nrf2

Cumulus cells were removed from MII oocytes, after which the oocytes were fixed, permeabilized, and blocked. Samples were incubated with the primary anti-Nrf2 antibody overnight at 4 °C, followed by incubation with a fluorescent secondary antibody in the dark and nuclear counterstaining. Images for all groups were acquired using identical laser power, gain, pinhole, and exposure settings. Background-corrected mean Nrf2 fluorescence intensity within the whole-oocyte region of interest was quantified using ImageJ (version 1.53t; NIH, Bethesda, MD, USA).

### 2.9. Gene Expression Analysis in Sheep Oocytes

#### 2.9.1. SMART-Seq2 Transcriptomic Analysis

After IVM, mature oocytes from the Control and MLT+GSH groups were collected for low-input transcriptomic sequencing. Oocytes were washed three times in HM199 and gently pipetted to remove residual cumulus cells, followed by three additional washes in DPBS. All procedures were completed at 37.5 °C within 15 min. Individual mature oocytes were transferred into RNase-free tubes containing 2.5 μL lysis buffer, immediately placed on ice, and stored at −80 °C. Three biological replicates were prepared per group and transported on dry ice to Lianchuan Biotechnology Co., Ltd. (Hangzhou, China) for SMART-seq2 sequencing and bioinformatic analysis. Differentially expressed genes were defined using |log2(fold change)| ≥ 1 and q < 0.05.

#### 2.9.2. RNA Preparation and Quantitative Real-Time PCR

Mature oocytes from each group were individually collected into 200 μL RNase-free tubes and stored at −80 °C. For analysis, 5 μL cell-lysis buffer was added to each sample, followed by centrifugation and lysis at 75 °C for 10 min. Genomic DNA was removed, and reverse transcription was performed at 37 °C for 15 min and 85 °C for 5 s to obtain cDNA. Quantitative real-time PCR (qRT-PCR) was performed using SYBR Green I with the following cycling program: initial denaturation at 95 °C for 30 s; 40 cycles of denaturation at 95 °C for 10 s, annealing at 60 °C for 30 s, and extension at 72 °C for 30 s. β-Actin was used as the reference gene, and relative target-gene expression was calculated using the 2^−ΔΔCt^ method. The primer sequences, amplicon sizes, and corresponding GenBank accession numbers are presented in Table 2.

### 2.10. Statistical Analysis

Fluorescence images were quantitatively analyzed using ImageJ 1.8.0. For embryo development, oocyte staining, and qRT-PCR assays, 30 oocytes per group were used in each of three independent biological replicates, resulting in a total of 90 oocytes per group. Data are presented as the mean ± SEM of the three independent replicates. For in vitro fertilization, the sperm concentration was standardized to 1 × 10^6^ sperm/mL.

Comparisons among the Control, MLT, GSH, and MLT+GSH groups, as well as among the Control, MLT+GSH, ML385, and MLT+GSH+ML385 groups, were performed using one-way ANOVA followed by Tukey’s multiple comparisons test. Because a factorial analysis of the MLT × GSH interaction was not performed, no statistical inference regarding synergistic effects between MLT and GSH was made. Accordingly, the effects of MLT+GSH co-supplementation were described in terms of the observed changes in individual endpoints rather than as a statistically defined synergistic effect. For multiple-group comparisons, different letters indicate significant differences between groups (*p* < 0.05), whereas groups sharing the same letter are not significantly different. For pairwise comparisons, ns indicates *p* ≥ 0.05, while *, **, ***, and **** indicate *p* < 0.05, *p* < 0.01, *p* < 0.001, and *p* < 0.0001, respectively. All statistical analyses were performed using GraphPad Prism(version 10.1.2; GraphPad Software, San Diego, CA, USA), with *p* < 0.05 considered statistically significant. Because fluorescence assays performed in different experimental modules were based on independently acquired batches, fluorescence intensities were interpreted as relative values within each corresponding experimental batch. Accordingly, fluorescence values were used only for within-assay group comparisons and were not directly compared across different figures.

Differential expression analysis of the SMART-seq2 transcriptomic data was performed using DESeq2. Differentially expressed genes were identified using thresholds of |log_2_FoldChange| > 1 and a false discovery rate (FDR) < 0.05.

### 2.11. Oocyte Source and Ethics Statement

Sheep ovaries were obtained from healthy ewes that had passed routine veterinary inspection at a licensed commercial slaughterhouse in Inner Mongolia, China. All ovaries were by-products of routine commercial slaughter, and no animals were slaughtered specifically for this study. Immediately after slaughter, the ovaries were collected, placed in physiological saline prewarmed to 37 °C, and transported to the laboratory within 2–3 h. Upon arrival, the ovaries were washed three times with physiological saline containing gentamicin, and those showing obvious pathological changes, tissue damage, or morphological abnormalities were excluded. Standardized procedures for sample collection, transportation, selection, and processing were consistently applied across different experimental batches and seasons to minimize variations in sample handling. Different experimental modules were conducted using independently numbered oocyte batches following the same standardized procedures. Fluorescence assays conducted in different experimental modules were performed using independent experimental batches to ensure that measurements were obtained separately under their respective experimental conditions. Detailed information on the experimental batches, acquisition dates, and imaging systems used for each assay is provided in Supplementary Table S1. The study was approved by the Animal Welfare and Research Ethics Committee of Inner Mongolia Agricultural University, and the experimental design and reporting followed the applicable ARRIVE 2.0 guidelines.

## 3. Results

### 3.1. MLT+GSH Co-Treatment Improves Sheep Oocyte Maturation

**Figure 1:**
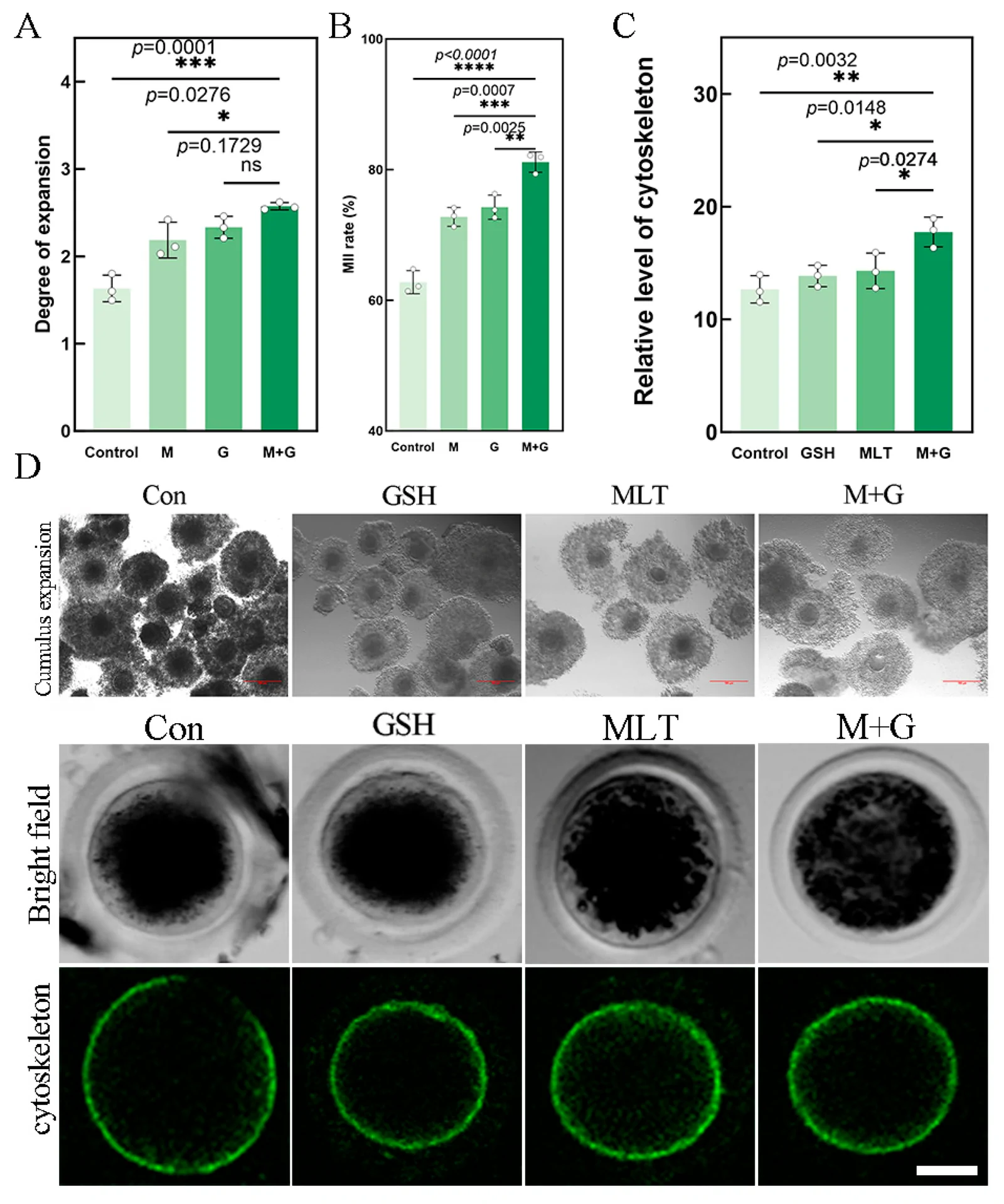
Co-treatment with MLT and GSH improves the maturation of sheep oocytes. (**A**) Cumulus expansion scores in the Control (Con), MLT (M), GSH (G), and MLT+GSH (M+G) groups; (**B**) first polar body extrusion (MII) rates; (**C**) relative fluorescence intensity of the cortical F-actin cytoskeleton; and (**D**) representative images of cumulus expansion and F-actin staining. Green fluorescence indicates F-actin organization in the oocyte cortex. Data are presented as the mean ± SEM; points overlaid on bars represent independent experimental replicates. Different lowercase letters above the bars indicate statistically significant differences whereas the same letters indicate no significant difference (ns). (* *p* < 0.05, ** *p* < 0.01, *** *p* < 0.001, and **** *p* < 0.0001); ns, not significant. Scale bars: 20 μm for stained oocytes and 100 μm for cumulus-expansion images. Fluorescence values are relative values derived within the corresponding experimental batch and are intended only for within-figure comparisons among treatment groups; they should not be directly compared with fluorescence values presented in other figures.

The effects of MLT, GSH, and their combined treatment on cumulus expansion, first polar body extrusion (MII rate), and cytoskeletal organization were evaluated in sheep oocytes. Both MLT and GSH alone increased the degree of cumulus expansion, whereas the MLT+GSH group exhibited the highest expansion score. The score in the MLT+GSH group was significantly higher than that in the Control and MLT groups (*p* = 0.0001 and *p* = 0.0276, respectively), but did not differ significantly from that in the GSH group (*p* = 0.1729; Figure 1A). Similarly, both single treatments increased the MII rate, while the MLT+GSH group showed a significantly higher MII rate than the Control, MLT, and GSH groups (*p* < 0.0001, *p* = 0.0007, and *p* = 0.0025, respectively; Figure 1B). F-actin staining revealed a distinct cortical distribution pattern in all groups, with the MLT+GSH group displaying the highest relative fluorescence intensity. This value was significantly greater than those observed in the Control, GSH, and MLT groups (*p* = 0.0032, *p* = 0.0148, and *p* = 0.0274, respectively; Figure 1C). Representative images further demonstrated a more continuous and uniform cortical F-actin distribution following MLT+GSH co-treatment (Figure 1D). Collectively, MLT+GSH co-treatment was associated with enhanced nuclear maturation and improved cortical cytoskeletal organization, although its effect on cumulus expansion was not significantly different from that of GSH alone.

### 3.2. MLT+GSH Co-Treatment Improves Redox Homeostasis and Mitochondrial Indices in Sheep Oocytes

**Figure 2:**
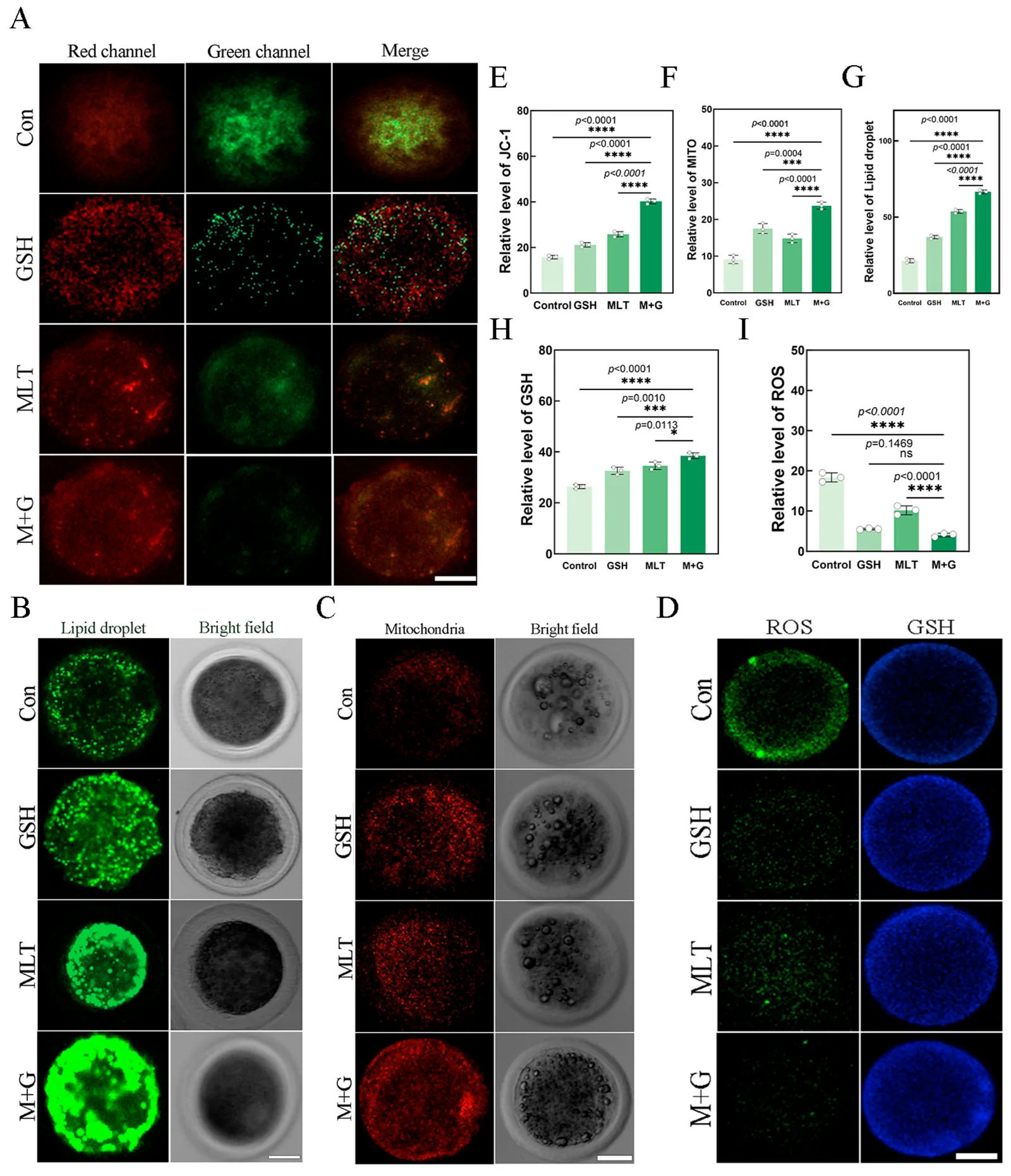
Co-treatment with MLT and GSH improves redox homeostasis and mitochondrial-associated indices in sheep oocytes. (**A**) Representative JC-1 fluorescence images for assessment of mitochondrial membrane potential; (**B**) representative lipid-droplet fluorescence and bright-field images; (**C**) representative Mito-Tracker fluorescence images; (**D**) representative ROS- and GSH-associated fluorescence images, in which green and blue fluorescence indicate ROS- and GSH-associated signals, respectively; (**E**) quantitative analysis of the JC-1 red-to-green fluorescence ratio; (**F**) Mito-Tracker fluorescence intensity; (**G**) lipid-droplet-associated fluorescence intensity; (**H**) GSH-associated fluorescence intensity; and (**I**) ROS-associated fluorescence intensity. Data are presented as the mean ± SEM; points overlaid on bars represent independent experimental replicates. Different asterisks above the bars indicate statistically significant differences. (* *p* < 0.05, *** *p* < 0.001, and **** *p* < 0.0001); whereas the same letters indicate no significant difference (ns). Scale bar = 20 μm. Fluorescence values are relative within each experimental batch and are intended only for within-figure comparisons; therefore, values should not be directly compared across figures.

To evaluate redox status and organelle-associated changes in sheep oocytes, intracellular ROS levels, GSH-associated fluorescence, mitochondrial membrane potential, MitoTracker fluorescence, and lipid droplet-associated fluorescence were examined. JC-1 staining showed that the red-to-green fluorescence ratio was increased by either MLT or GSH treatment alone and reached the highest level in the MLT+GSH group, which was significantly higher than those in the Control, GSH, and MLT groups (all *p* < 0.0001; Figure 2A,E). MitoTracker fluorescence was also highest in the MLT+GSH group and was significantly greater than that in the Control (*p* < 0.0001), GSH (*p* = 0.0004), and MLT (*p* < 0.0001) groups (Figure 2C,F). Lipid droplet-associated fluorescence increased following MLT or GSH treatment and was highest in the MLT+GSH group, with significant differences compared with the Control, GSH, and MLT groups (all *p* < 0.0001; Figure 2B,G). GSH-associated fluorescence was elevated following either MLT or GSH treatment alone and further increased in the MLT+GSH group, which was significantly higher than the Control (*p* < 0.0001), GSH (*p* = 0.0010), and MLT (*p* = 0.0113) groups (Figure 2D,H). In contrast, ROS-associated fluorescence was lower in all treatment groups than in the Control group, with the lowest value observed in the MLT+GSH group. The MLT+GSH group differed significantly from the Control and MLT groups (both *p* < 0.0001), whereas no significant difference was detected between the MLT+GSH and GSH groups (*p* = 0.1469; Figure 2D,I). Collectively, MLT+GSH co-treatment was associated with reduced ROS accumulation together with increased GSH-associated fluorescence, mitochondrial membrane potential, mitochondrial-associated fluorescence, and lipid droplet-associated fluorescence during sheep oocyte maturation.

### 3.3. MLT+GSH Co-Treatment Promotes Early Embryonic Development and Reduces Apoptosis-Related Readouts

**Figure 3:**
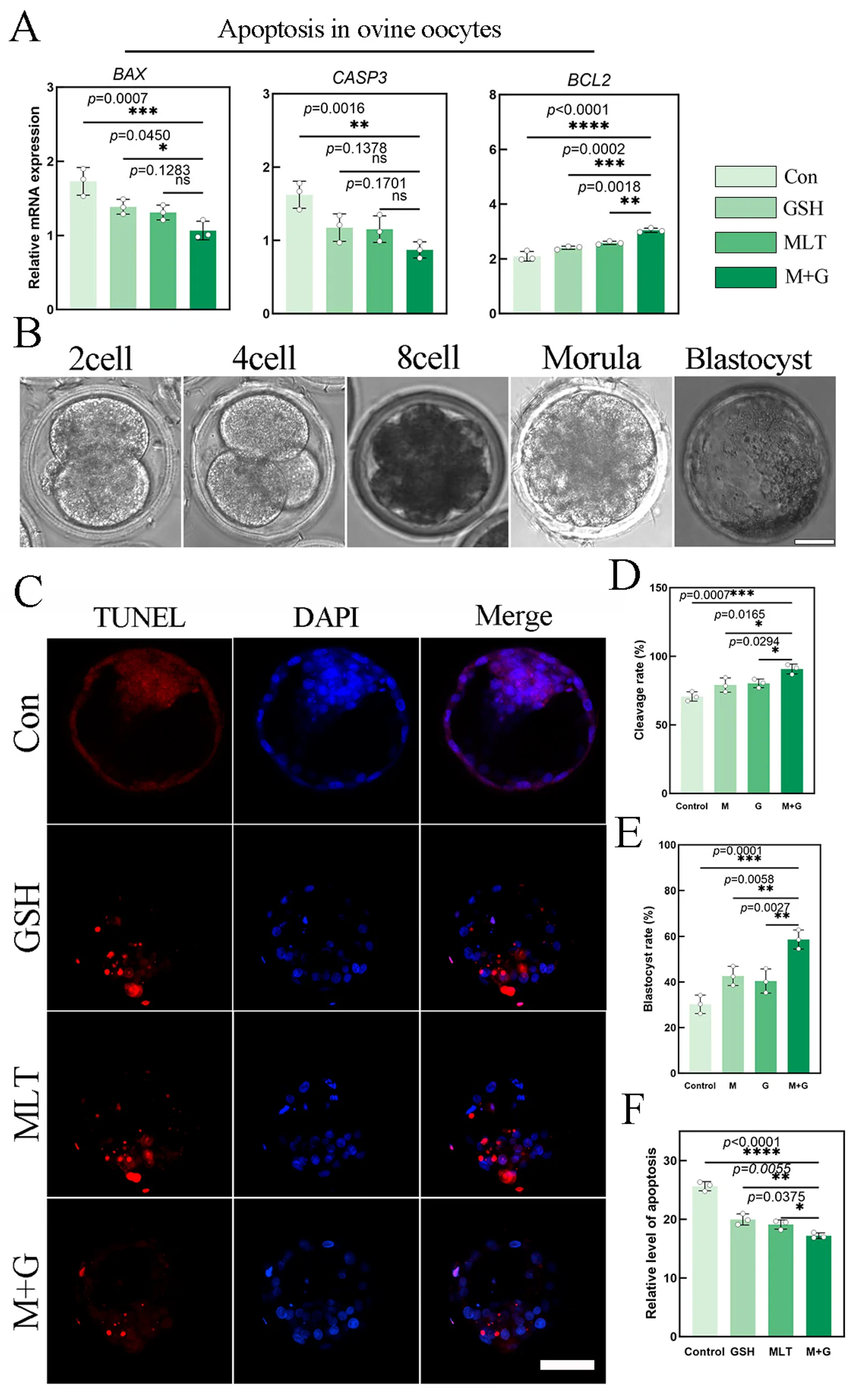
Co-treatment with MLT and GSH promotes early embryonic development and reduces apoptosis-related readouts. (**A**) Relative mRNA expression of BAX, CASP3, and BCL2; (**B**) representative images of embryos at the 2-cell, 4-cell, 8-cell, morula, and blastocyst stages after in vitro fertilization; (**C**) representative TUNEL images of blastocysts, in which red fluorescence indicates TUNEL-positive nuclei and blue fluorescence indicates DAPI-stained nuclei; (**D**) cleavage rates; (**E**) blastocyst formation rates; and (**F**) quantitative analysis of apoptosis at the blastocyst stage. Data are presented as the mean ± SEM; points overlaid on bars represent independent experimental replicates. Different asterisks above the bars indicate statistically significant differences (* *p* < 0.05, ** *p* < 0.01, *** *p* < 0.001, and **** *p* < 0.0001); whereas the same letters indicate no significant difference (ns). Scale bar = 20 μm.

Embryonic developmental competence and apoptosis-related changes were evaluated following in vitro fertilization. Both MLT and GSH alone increased cleavage and blastocyst formation rates, whereas the MLT+GSH group showed the highest developmental outcomes. Compared with the MLT+GSH group, cleavage rates were significantly lower in the Control, MLT, and GSH groups (*p* = 0.0007, *p* = 0.0165, and *p* = 0.0294, respectively; Figure 3B,D). Similarly, the blastocyst formation rate was significantly higher in the MLT+GSH group than in the Control, MLT, and GSH groups (*p* = 0.0001, *p* = 0.0058, and *p* = 0.0027, respectively; Figure 3B,E). TUNEL analysis showed reduced apoptosis-associated fluorescence in the MLT-, GSH-, and MLT+GSH-treated blastocysts relative to the Control group, with the lowest apoptotic level observed in the MLT+GSH group. The MLT+GSH group differed significantly from the Control, GSH, and MLT groups (*p* < 0.0001, *p* = 0.0055, and *p* = 0.0375, respectively; Figure 3C,F). Consistently, qRT-PCR analysis showed that MLT+GSH treatment was associated with lower expression of the pro-apoptotic genes BAX and CASP3 and higher expression of the anti-apoptotic gene BCL2. BAX expression was significantly higher in the Control and GSH groups than in the MLT+GSH group (*p* = 0.0007 and *p* = 0.0450, respectively), whereas the difference between the MLT and MLT+GSH groups was not significant (*p* = 0.1283). CASP3 expression was significantly higher in the Control group than in the MLT+GSH group (*p* = 0.0016), while no significant differences were observed between the MLT+GSH group and either the GSH or MLT group (*p* = 0.1378 and *p* = 0.1701, respectively). In contrast, BCL2 expression was significantly higher in the MLT+GSH group than in the Control, GSH, and MLT groups (*p* < 0.0001, *p* = 0.0002, and *p* = 0.0018, respectively; Figure 3A). Collectively, MLT+GSH co-treatment was associated with enhanced embryonic developmental competence, reduced blastocyst apoptosis, and a more favorable apoptosis-related gene-expression profile.

### 3.4. Transcriptomic Changes Associated with MLT+GSH Co-Treatment in Sheep Oocytes

**Figure 4:**
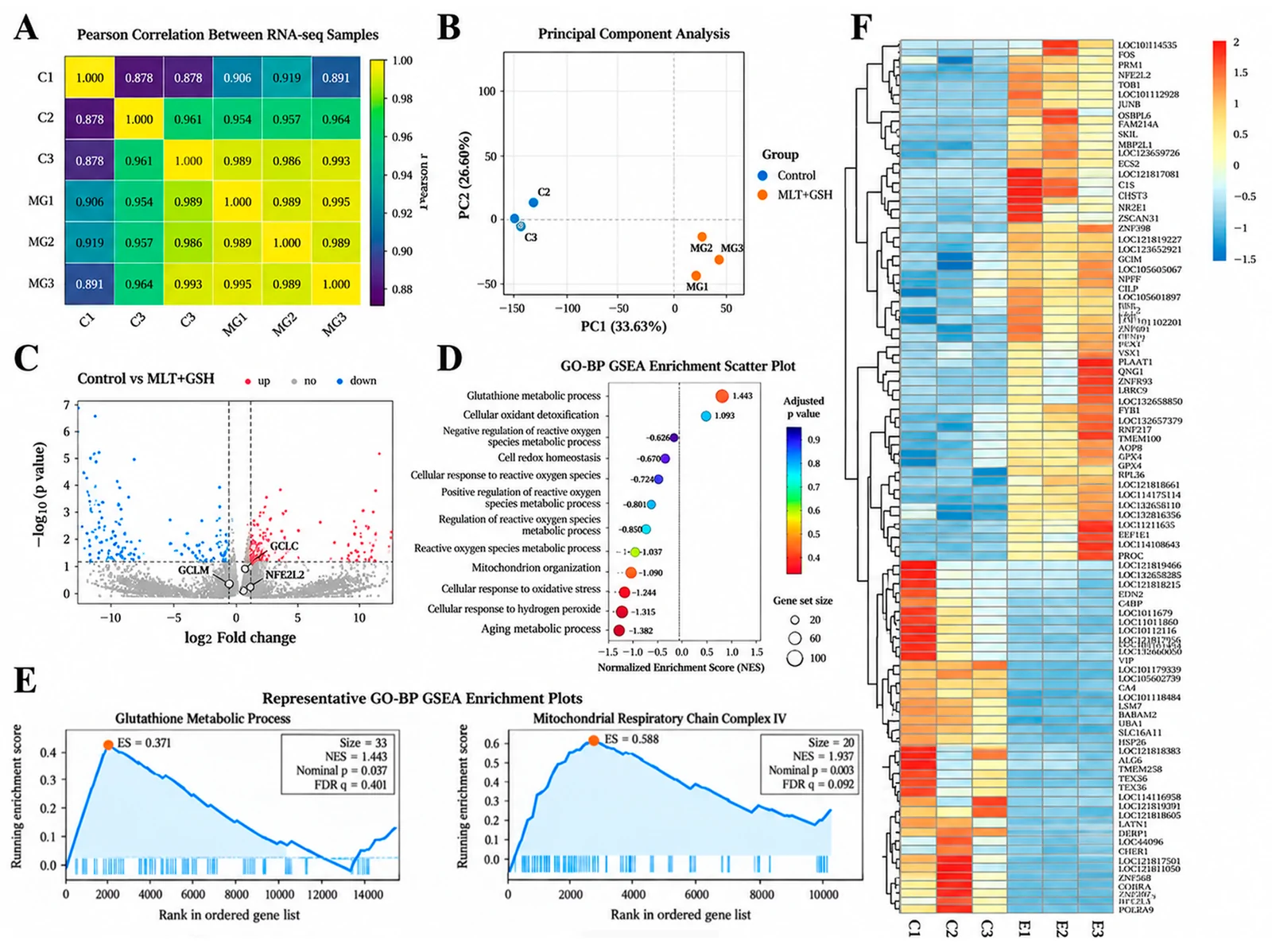
(**A**) Pearson correlation heatmap among RNA-seq samples; (**B**) principal component analysis (PCA) based on transcriptomic expression profiles; (**C**) volcano plot of differentially expressed genes between the control group and the MLT+GSH group; (**D**) GO Biological Process (GO-BP) GSEA enrichment results; (**E**) representative GO-BP GSEA enrichment curves; (**F**) hierarchical clustering heatmap of gene expression related to Nrf2/GSH/redox status.

To characterize transcriptomic changes in sheep oocytes following combined MLT and GSH treatment, RNA-seq analysis was performed in the Control and MLT+GSH groups. Sample correlation analysis showed generally high correlations among the six samples. Within the Control group, the Pearson correlation coefficients between C1 and C2 and between C1 and C3 were both 0.878, whereas correlations among the three MLT+GSH samples ranged from 0.989 to 0.995 (Figure 4A). Principal component analysis (PCA) showed that the two groups were primarily separated along PC1, with PC1 and PC2 accounting for 33.63% and 26.60% of the total variance, respectively (Figure 4B). Sequencing depth, Q30 values, GC content, genome mapping rates, and the numbers of detected genes were generally stable across samples, with no obvious sequencing-quality outliers identified.

Differential expression analysis revealed a range of transcriptional changes between the Control and MLT+GSH groups (Figure 4C). Gene set enrichment analysis based on the ranked whole-transcriptome dataset further identified varying degrees of enrichment in several GO biological process gene sets related to glutathione metabolism, oxidative-stress responses, reactive oxygen species metabolism, cellular redox homeostasis, and mitochondrial function (Figure 4D). Among these, the glutathione metabolic process gene set showed positive enrichment in the MLT+GSH direction (NES = 1.443, nominal *p* = 0.037, FDR q = 0.401), while mitochondrial respiratory chain complex IV was also positively enriched (NES = 1.937, nominal *p* = 0.003, FDR q = 0.092; Figure 4E).

Hierarchical clustering of Nrf2/GSH/redox-related genes revealed distinct expression patterns between the Control and MLT+GSH groups (Figure 4F). Among the six candidate genes examined in detail—NFE2L2, NQO1, HMOX1, GCLC, GCLM, and GPX4—NFE2L2, NQO1, and GCLC exhibited positive expression trends, whereas GCLM showed a relatively small change with a slight negative trend.

### 3.5. OocytesMLT+GSH Co-Treatment Enhances Nrf2-Associated Antioxidant Responses in Sheep Oocytes

**Figure 5:**
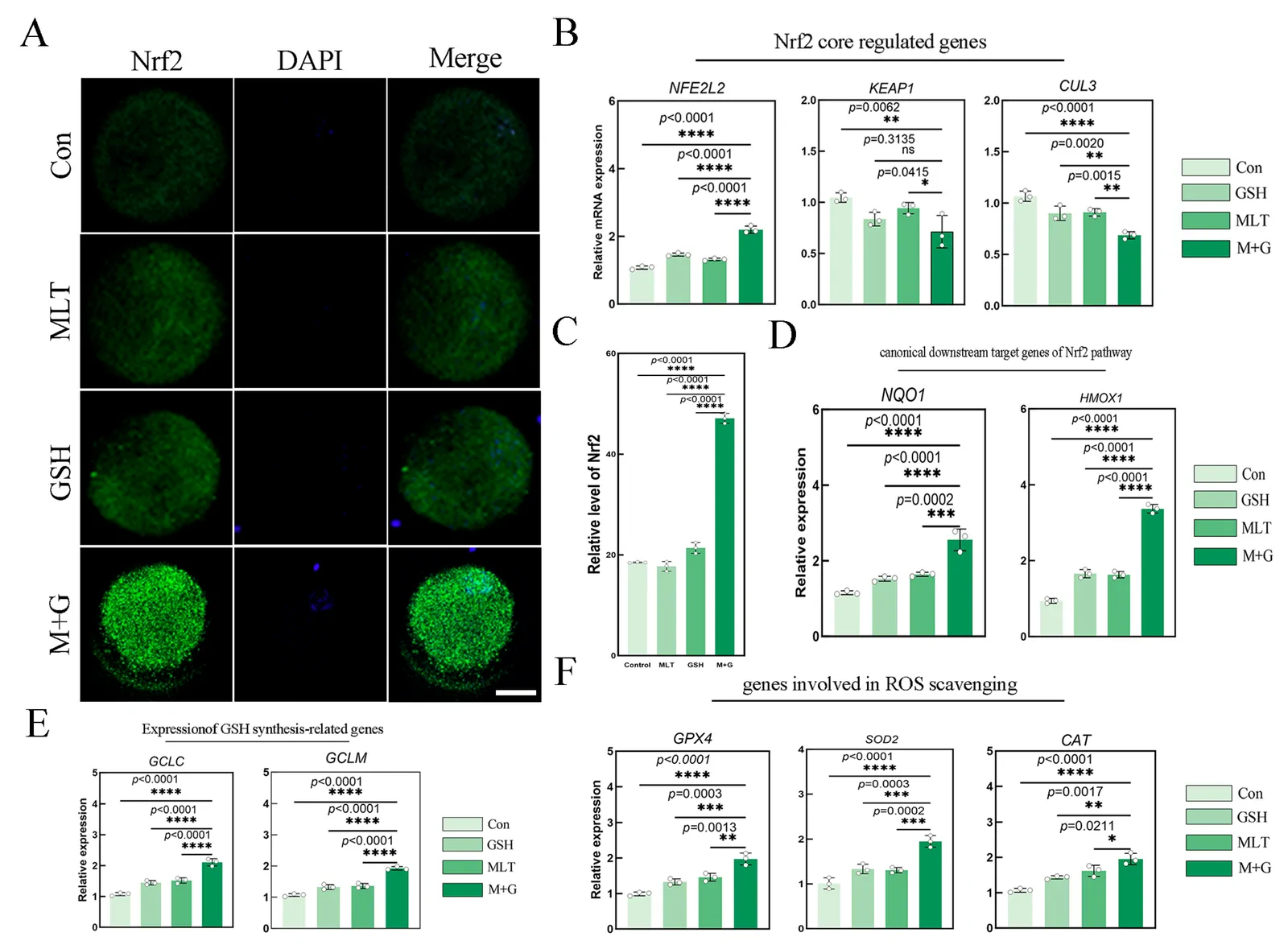
Co-treatment with MLT and GSH enhances Nrf2-associated antioxidant responses in sheep oocytes. (**A**) Representative Nrf2 immunofluorescence images in the Control, GSH, MLT, and MLT+GSH groups; green fluorescence indicates Nrf2 and blue fluorescence indicates DAPI-stained nuclei; (**B**) relative mRNA expression of NFE2L2, KEAP1, and CUL3; (**C**) quantitative analysis of whole-oocyte Nrf2 fluorescence intensity; (**D**) relative mRNA expression of the canonical Nrf2 target genes NQO1 and HMOX1; (**E**) relative mRNA expression of the glutathione-synthesis genes GCLC and GCLM; and (**F**) relative mRNA expression of the ROS-detoxification-related genes GPX4, SOD2, and CAT. Data are presented as the mean ± SEM; points overlaid on bars represent independent experimental replicates. Different asterisks above the bars indicate statistically significant differences (* *p* < 0.05, ** *p* < 0.01, *** *p* < 0.001, and **** *p* < 0.0001); whereas the same letters indicate no significant difference (ns). Scale bar = 20 μm. Nrf2 fluorescence values are relative within each experimental batch and are intended only for within-figure comparisons, not for direct comparison across figures.

To investigate the effects of MLT and GSH on Nrf2-associated antioxidant responses, whole-oocyte Nrf2 immunofluorescence intensity and the expression of related genes were analyzed. Nrf2 fluorescence signals were detectable in oocytes from all groups, and the relative whole-oocyte Nrf2 fluorescence intensity was highest in the MLT+GSH group, which was significantly greater than those in the Control, MLT, and GSH groups (all *p* < 0.0001; Figure 5A,C). qRT-PCR analysis showed that MLT+GSH treatment significantly increased NFE2L2 expression while decreasing the expression levels of KEAP1 and CUL3 (Figure 5B). KEAP1 expression was significantly lower in the MLT+GSH group than in the Control and MLT groups, whereas no significant difference was observed between the MLT+GSH and GSH groups (*p* = 0.3135). Further analysis of Nrf2-related downstream genes showed that NQO1 and HMOX1 expression levels were significantly higher in the MLT+GSH group than in the Control, MLT, and GSH groups (Figure 5D). Likewise, the GSH synthesis-related genes GCLC and GCLM were significantly upregulated in the MLT+GSH group (Figure 5E). In addition, GPX4, SOD2, and CAT expression levels were significantly higher in the MLT+GSH group than in the Control, GSH, and MLT groups (all *p* < 0.05; Figure 5F). Overall, MLT+GSH co-treatment was associated with increased whole-oocyte Nrf2 immunofluorescence intensity and coordinated changes in the expression of multiple Nrf2-related antioxidant genes.

### 3.6. Nrf2 Inhibition Attenuates the Protective Effects of MLT+GSH Co-Treatment in Sheep Oocytes

**Figure 6:**
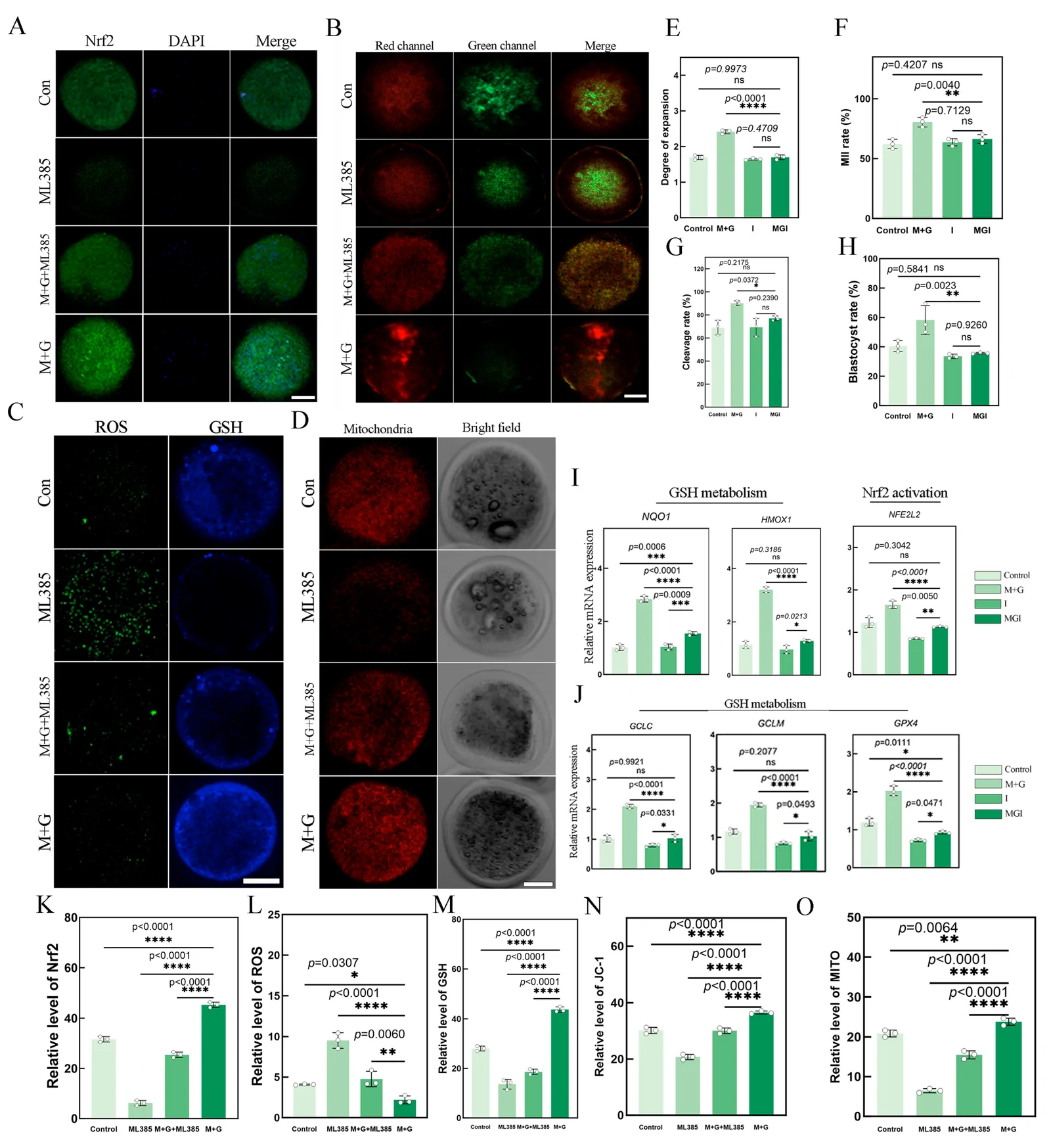
Nrf2 inhibition attenuates the protective effects of MLT+GSH co-treatment in sheep oocytes. (**A**) Representative Nrf2 immunofluorescence images in the Control, MLT+GSH, ML385, and MLT+GSH+ML385 groups; green fluorescence indicates Nrf2 and blue fluorescence indicates DAPI-stained nuclei; (**B**) representative JC-1 fluorescence images; (**C**) representative ROS- and GSH-associated fluorescence images; (**D**) representative Mito-Tracker fluorescence images; (**E**) cumulus expansion; (**F**) first polar body extrusion (MII) rate; (**G**) cleavage rate; (**H**) blastocyst formation rate; (**I**) relative mRNA expression of NQO1, HMOX1, and NFE2L2; (**J**) relative mRNA expression of GCLC, GCLM, and GPX4; (**K**) Relative whole-oocyte Nrf2 fluorescence intensity; (**L**) ROS-associated fluorescence intensity; (**M**) GSH-associated fluorescence intensity; (**N**) JC-1 red-to-green fluorescence ratio; and (**O**) Mito-Tracker fluorescence intensity. Data are presented as the mean ± SEM; points overlaid on bars represent independent experimental replicates. Different asterisks above the bars indicate statistically significant differences (* *p* < 0.05, ** *p* < 0.01, *** *p* < 0.001, and **** *p* < 0.0001); whereas the same letters indicate no significant difference (ns). Scale bar = 20 μm. Fluorescence values are relative within each experimental batch and are intended only for within-figure comparisons, not for direct comparison across figures.

To further evaluate the potential involvement of Nrf2-related regulation in the effects of MLT+GSH co-treatment, Nrf2 immunofluorescence, redox status, mitochondrial function, oocyte maturation, and embryonic developmental outcomes were compared among the Control, ML385, MLT+GSH+ML385, and MLT+GSH groups. Nrf2 immunofluorescence analysis showed that the MLT+GSH group exhibited the highest relative fluorescence intensity, whereas the signal was significantly reduced in the MLT+GSH+ML385 group following ML385 treatment (*p* < 0.0001; Figure 6A,K).

Analysis of redox-related parameters showed that ROS levels were lowest in the MLT+GSH group and were significantly increased in the MLT+GSH+ML385 group (*p* = 0.0060). The ML385-alone group exhibited the highest ROS level, which was significantly higher than that in the MLT+GSH group (*p* < 0.0001; Figure 6C,L). In contrast, GSH-associated fluorescence was highest in the MLT+GSH group, decreased after ML385 addition, and was lowest in the ML385-alone group (Figure 6C,M). JC-1 analysis showed that the mitochondrial membrane potential-related index was highest in the MLT+GSH group, significantly decreased in the MLT+GSH+ML385 group, and was lowest in the ML385-alone group (Figure 6B,N). A similar pattern was observed for MitoTracker fluorescence, with the highest mitochondrial-associated signal in the MLT+GSH group, a lower signal in the MLT+GSH+ML385 group, and the lowest level in the ML385-alone group (Figure 6D,O).

With respect to oocyte maturation and subsequent embryonic development, the MLT+GSH group generally showed higher levels of cumulus expansion, MII rate, cleavage rate, and blastocyst formation. Following ML385 treatment, these indices were reduced to varying degrees in the MLT+GSH+ML385 group, with significant decreases in the MII rate, cleavage rate, and blastocyst formation rate relative to the MLT+GSH group (Figure 6E–H). Although the ML385-alone group did not differ significantly from the Control group for some maturation and developmental endpoints, marked changes were observed in several redox- and mitochondria-related parameters, indicating that the effects of ML385 were not uniform across all measured outcomes.

Further qRT-PCR analysis showed that, compared with the MLT+GSH group, the MLT+GSH+ML385 group exhibited reduced expression of NFE2L2 and the Nrf2-related downstream antioxidant genes NQO1, HMOX1, GCLC, GCLM, and GPX4, with most comparisons reaching statistical significance (*p* < 0.05; Figure 6I,J). Overall, ML385 intervention partially attenuated the MLT+GSH-associated increase in Nrf2 immunofluorescence, elevation of GSH-related fluorescence, reduction in ROS levels, improvement in mitochondrial function, and enhancement of maturation and embryonic developmental outcomes, although the magnitude of these effects varied among the different endpoints.

## 4. Discussion

The present study systematically evaluated the effects of combined melatonin (MLT) and glutathione (GSH) supplementation on the in vitro maturation (IVM) and subsequent developmental competence of sheep oocytes and further explored the potential regulatory basis from the perspectives of redox homeostasis, mitochondrial status, and Nrf2-associated antioxidant responses. Overall, MLT+GSH co-treatment produced a favorable profile across multiple maturation and redox-related endpoints, including enhanced cumulus expansion and first polar body extrusion, reduced ROS-associated fluorescence, increased GSH-associated fluorescence, improved mitochondrial indices, and enhanced subsequent embryonic development [12,13]. SMART-seq2 profiling, antioxidant-related gene expression, and pharmacological intervention with ML385 further indicated that these phenotypic changes were accompanied by enhanced Nrf2-associated antioxidant responses, whereas inhibition of Nrf2 attenuated some of the protective effects [14]. A major contribution of this study is the application of combined MLT and GSH supplementation to sheep oocyte IVM and the integrated evaluation of a biological continuum encompassing redox homeostasis, mitochondrial status, oocyte maturation, and subsequent embryonic development. These findings extend previous studies focusing predominantly on individual antioxidants and provide new evidence for combined antioxidant regulation during oocyte IVM.

MLT and GSH are both important components of cellular antioxidant defense but exert their effects through partly distinct mechanisms. MLT can directly scavenge reactive species while also modulating endogenous antioxidant responses and maintaining mitochondrial homeostasis, thereby protecting oocytes against oxidative injury during maturation [15,16]. GSH, in contrast, is one of the major intracellular non-enzymatic antioxidants and serves as an essential reducing substrate for glutathione-dependent antioxidant reactions. Adequate GSH availability is closely associated with oocyte maturation, fertilization, and early embryonic development [17]. These distinct properties provide a biological rationale for their combined application: MLT may help limit oxidative burden, whereas GSH contributes to maintaining intracellular reducing capacity. In the present study, MLT+GSH treatment was associated with reduced ROS accumulation, enhanced GSH-related signals, and improvements in multiple maturation and developmental endpoints. Compared with previous studies examining MLT or GSH individually, our findings suggest that simultaneous modulation of oxidative burden and cellular reducing capacity may represent an effective strategy for maintaining a more favorable redox environment during IVM [18].

Nrf2-associated antioxidant regulation may represent an important molecular link between MLT+GSH supplementation and improved redox homeostasis. Nrf2, encoded by NFE2L2, is an important transcriptional regulator of cellular responses to oxidative stress and is associated with the regulation of multiple genes involved in antioxidant defense and glutathione metabolism, including NQO1, HMOX1, GCLC, GCLM, and GPX4 [19]. Nrf2-associated antioxidant responses have also been implicated in the maintenance of oocyte redox homeostasis and developmental competence [20]. In the present study, MLT+GSH treatment increased whole-oocyte Nrf2 immunofluorescence intensity and was accompanied by changes in NFE2L2 and several antioxidant-related genes, consistent with the observed reduction in ROS-associated fluorescence and increase in GSH-associated fluorescence. SMART-seq2 analysis further revealed broad transcriptional changes associated with cellular metabolism, stress responses, and homeostatic regulation following co-treatment. Together, these observations suggest that MLT+GSH supplementation may influence not only the immediate antioxidant environment but also endogenous antioxidant regulatory responses in sheep oocytes.

The marked increase in BODIPY 493/503-associated fluorescence following MLT+GSH treatment indicates altered intracellular lipid-droplet accumulation during oocyte maturation. Increased lipid storage may provide metabolic substrates for subsequent energy production, but excessive lipid accumulation may also reflect altered lipid mobilization or β-oxidation and has been associated with reduced cryotolerance in lipid-rich livestock oocytes. Therefore, the increased lipid-droplet signal should not be interpreted as unequivocally beneficial. Notably, the increase in lipid-associated fluorescence occurred together with enhanced GSH-associated fluorescence, reduced ROS accumulation, and increased GPX4 expression. Because GPX4 participates in GSH-dependent detoxification of lipid hydroperoxides, these coordinated changes may indicate remodeling of lipid metabolism together with altered lipid redox regulation. However, lipid peroxidation was not directly measured in the present study; therefore, future studies using BODIPY 581/591 C11, MDA, and β-oxidation-related markers will be required to determine the functional significance of the increased lipid-droplet content.

Within this antioxidant network, GCLC and GCLM are key components involved in GSH biosynthesis and contribute to the maintenance of intracellular reducing capacity. NQO1 and HMOX1 are representative molecules associated with Nrf2-regulated antioxidant and stress responses, whereas GPX4 utilizes GSH to reduce lipid hydroperoxides and thereby contributes to the maintenance of membrane redox homeostasis [21]. In the present study, changes in the expression of these antioxidant-related genes following MLT+GSH treatment were accompanied by increased GSH-associated fluorescence and reduced ROS accumulation. This concordance suggests that the effects of combined supplementation may involve coordinated regulation of GSH metabolism and peroxide detoxification rather than solely the direct scavenging of reactive species. Such modulation of endogenous antioxidant responses may contribute to the maintenance of redox homeostasis during oocyte maturation. Nevertheless, the present findings support the involvement of an Nrf2-associated antioxidant response, rather than establishing Nrf2 as the sole determinant of the protective effects.

The relationship between redox homeostasis and mitochondrial status provides an additional biological context for the improved developmental phenotype. Mitochondria are not only a major source of intracellular ROS but also provide the energy required for meiotic progression, cytoskeletal remodeling, and early embryonic development [22]. Excessive ROS can disrupt mitochondrial homeostasis, while mitochondrial dysfunction can further exacerbate oxidative stress. In the present study, improvements in ROS- and GSH-related parameters following MLT+GSH supplementation were accompanied by favorable changes in mitochondrial-related indices and improved cumulus expansion and first polar body extrusion. These concordant changes suggest that maintaining a more favorable redox environment during IVM may help preserve mitochondrial status and thereby support meiotic maturation and cellular organization.

Importantly, the beneficial effects observed during IVM extended to subsequent embryonic development. MLT+GSH supplementation increased cleavage and blastocyst formation rates and was accompanied by favorable changes in apoptosis-related markers, including BAX, CASP3, and BCL2 [23]. These findings suggest that the effects of combined supplementation are not restricted to meiotic maturation but may also improve the maternal competence of oocytes to support early embryogenesis. Because the redox environment, mitochondrial status, and intracellular molecular reserves established during oocyte maturation can influence post-fertilization development, improving the IVM environment through combined antioxidant supplementation may provide a more favorable maternal foundation for subsequent embryonic development.

Pharmacological intervention with ML385 provided additional functional support for the potential involvement of Nrf2-associated antioxidant regulation. ML385 treatment attenuated several MLT+GSH-associated changes in antioxidant-related gene expression, redox status, mitochondrial-related indices, and maturation phenotypes [24]. However, ML385 did not completely abolish all beneficial effects of the combined treatment. These observations suggest that Nrf2-associated regulation may contribute to, rather than exclusively mediate, the protective effects of MLT+GSH. This interpretation is also consistent with the pleiotropic antioxidant properties of MLT and GSH, which are likely to involve multiple pathways associated with oxidative stress, mitochondrial homeostasis, and cellular adaptation.

The present study has several limitations. First, although the Control, MLT, GSH, and MLT+GSH groups allowed direct comparison of individual and combined supplementation, the statistical analysis did not formally evaluate an MLT × GSH interaction. Accordingly, the findings support an enhanced protective effect of co-treatment but do not establish a statistically defined synergistic interaction. Second, the involvement of Nrf2 was evaluated primarily through whole-oocyte Nrf2 immunofluorescence intensity, antioxidant-related gene expression, and pharmacological inhibition with ML385. Nrf2 nuclear-to-cytoplasmic distribution was not quantitatively assessed in the present study. Therefore, more pathway-specific molecular approaches would further clarify the precise contribution of Nrf2-associated regulation to the effects of combined supplementation.

In conclusion, combined MLT and GSH supplementation improved the redox status of sheep oocytes during IVM and was accompanied by favorable changes in mitochondrial status, oocyte maturation, and subsequent embryonic developmental competence. Transcriptomic profiling, antioxidant-related gene expression, and ML385 intervention further suggest that Nrf2-associated antioxidant responses may contribute to these protective effects [25]. The present study establishes a functional link among redox homeostasis, mitochondrial status, oocyte maturation, and developmental competence from the perspective of combined antioxidant regulation and extends our understanding of Nrf2-associated antioxidant responses during sheep oocyte IVM. From an applied perspective, combined MLT and GSH supplementation may represent a promising strategy for optimizing the antioxidant environment of livestock IVM systems, thereby providing a theoretical and experimental basis for improving oocyte quality and in vitro embryo production efficiency.

## 5. Conclusions

Co-treatment with MLT and GSH improved the in vitro maturation and subsequent developmental competence of sheep oocytes. The treatment reduced ROS-associated fluorescence, enhanced GSH-associated fluorescence, supported mitochondrial indices, promoted cumulus expansion and first polar body extrusion, and improved cleavage and blastocyst formation while reducing apoptosis-related readouts. Co-treatment was also associated with increased Nrf2 immunofluorescence intensity and coordinated changes in NFE2L2, NQO1, HMOX1, GCLC, GCLM, and GPX4 expression. Pharmacological inhibition with ML385 attenuated these improvements, suggesting that Nrf2-associated antioxidant regulation may contribute to these effects. Thus, MLT+GSH co-treatment may provide a useful approach for improving redox homeostasis and developmental competence under standard IVM conditions, although additional pathway-specific validation is required.

## Figures and Tables

**Table 1:** Experimental Groups.

Code	Group	Additives to the IVM Medium (Concentration)
C	Control	No exogenous additive (Section 3.1, Section 3.2, Section 3.3, Section 3.4 and Section 3.5); DMSO vehicle control matched to the final DMSO concentration in ML385-treated groups (Section 3.6).
M	MLT	10^−9^ mol/L melatonin
G	GSH	2 mmol/L glutathione
MG	MLT+GSH	10^−9^ mol/L melatonin + 2 mmol/L glutathione
I	ML385	5 μmol/L ML385
MGI	MLT+GSH+ML385	10^−9^ mol/L melatonin + 2 mmol/L glutathione + 5 μmol/L ML385

**Table 2:** Primer information for each target gene.

Gene	Primer Sequences (5′-3′)		Size (bp)	GenBank
	Forward	Reverse		Accession Number
NFE2L2	ACACCATCAGAAGCCAGTGG	GCTGCATGCAGTCATCGAAG	163	XM_015093345.4
KEAP1	TGGAGACAGAAACGTGGACG	CAGGTGTCTGTGTCTGGGTC	157	XM_027969637.3
CUL3	CACTCCAGCATCACATGGGT	TGCACCTCCAACACCAACTT	95	XM_042244311.2
CAT	CCAGATGGACACAGGCACAT	GGCCGTAGTCAGGATCTTCG	169	XM_060400055.1
CASP3	AGCAAATCAGTGGACTCCGG	GCTCGTGAAGGTTTCCCTGA	171	XM_060406953.1
GCLC	GACCCGCTGACTCTGTTTGA	TCGAAGTCCGTCAGCTGAAC	179	XM_015102643.4
GCLM	TGGAGTTGCACAGCTGGATT	GCTGCTCCAACTGGGTTTTG	173	XM_027972220.2
GPX4	ATGAAAGTCCAGCCCAAGGG	GACCATACCGCTTCACCACA	106	XM_060416444.1
BCL2	ATCCTGGGGCGTGAGAAAAG	AGAATCCCATGGACGGAGGA	139	XM_012103831.5
BAX	CATGGGCTGGACATTGGACT	AAAGTAGGAGAGGAGGCCGT	88	XM_027978592.3
HMOX1	TTCCTGTTCCTGCAGCAGAG	GGAGACTTCAGACCCAAGGC	85	XM_027967703.3
NQO1	AGGATGGAAGAAACGCCTGG	GCTCATCCTGCACCTCCTTT	122	XM_004015102.6
SOD2	CCGTCAGCCTTACACCAAGT	CAAGCCACGCTCAGAAACAC	112	NM_001280703.1

## Data Availability

The transcriptome datasets generated and analyzed during the current study have been deposited in Figshare and are publicly available at https://doi.org/10.6084/m9.figshare.33942391.

## Supplementary Materials

The following supporting information can be downloaded at: https://www.mdpi.com/article/10.3390/biology15201858/s1. Table S1: RNA-seq Quality Control and Mapping Statistics. Table S2: Complete RNA-seq Differential Gene Expression Results Between the Control and MLT+GSH Groups. Figure S1: Reference-based schematic for ML385 dose selection.
